# [Corrigendum] Effect of SDF-1/CXCR4 axis on the migration of transplanted bone mesenchymal stem cells mobilized by erythropoietin toward lesion sites following spinal cord injury

**DOI:** 10.3892/ijmm.2026.5909

**Published:** 2026-07-01

**Authors:** Jun Li, Weichun Guo, Min Xiong, Heng Han, Jie Chen, Dan Mao, Bing Tang, Hualong Yu, Yun Zeng

Int J Mol Med 36: 1205-1214, 2015; DOI: 10.3892/ijmm.2015.2344

Following the publication of this paper, it was drawn to the Editor's attention by a concerned reader that, regarding the experiments showing the localization of fluorescently-labeled cells transplanted into the injured spinal cord in Fig. 4 on p. 1210, the fluorescence images shown in Fig. 4A and 4B contained an overlapping section, such that data which were intended to have shown the results of differently performed experiments appeared to have been derived from the same original source.

Upon contacting the authors about these issues, they have realized that images featured in Fig. 4 of this article were inadvertently assembled incorrectly. The revised version of Fig. 4, now featuring data from a repeated experiment, is shown below [note that the original GFP-conjugated green secondary antibody was replaced with Alexa Fluor 594 (Cy3) red fluorescent secondary antibody, and the description has been revised in the figure legend accordingly]. The authors wish to emphasize that the error made in assembling the data in this figure did not affect the overall conclusions reported in the paper. The authors are grateful to the Editor of *International Journal of Molecular Medicine* for granting them this opportunity to publish a Corrigendum, and apologize to both the Editor and the readership for any inconvenience caused.

## Figures and Tables

**Figure 4:**
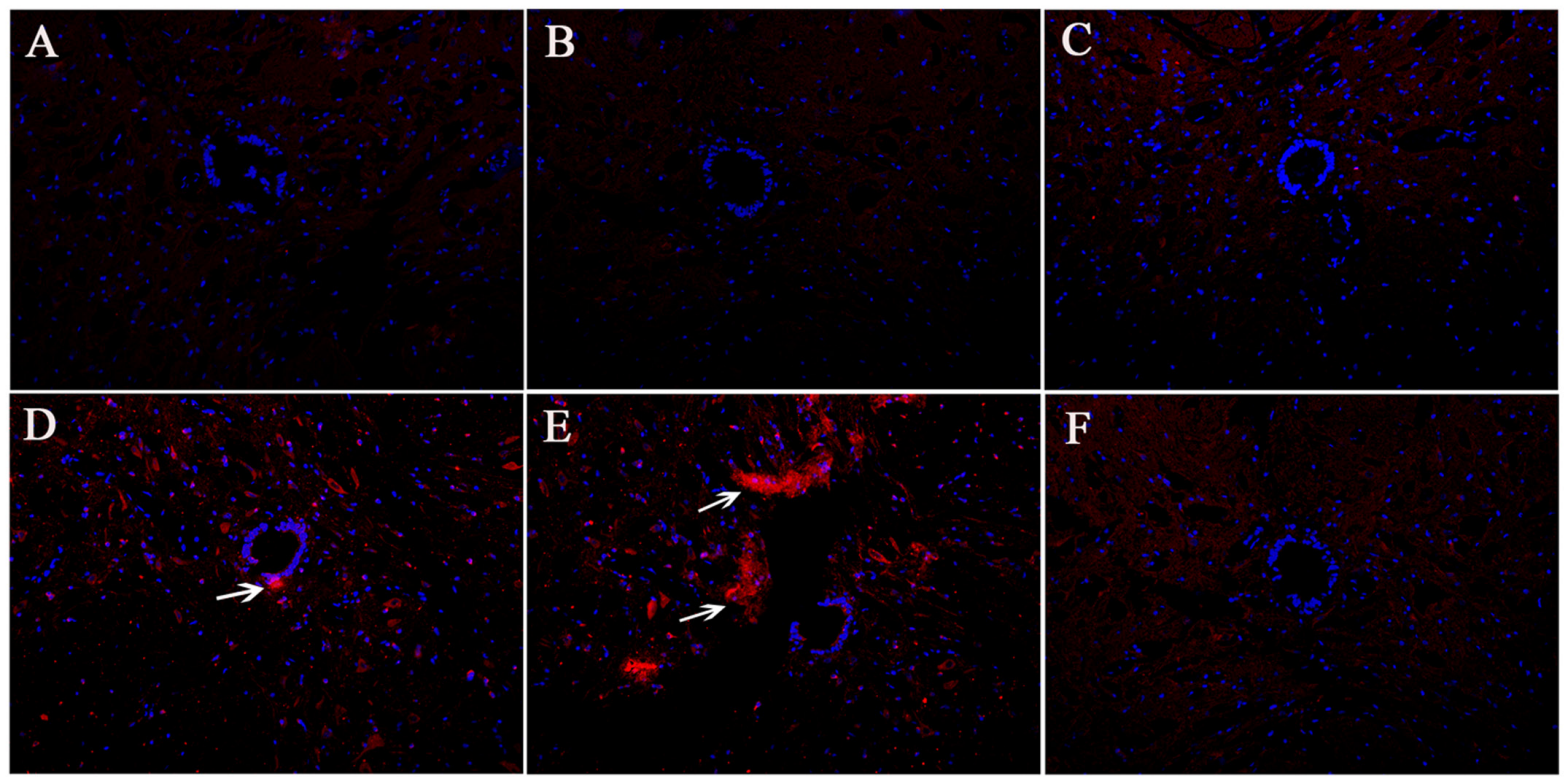
Localization of fluorescently-labeled cells transplanted into the injured spinal cord. (A) The sham-operated group, (B) model control, (C) EPO, (D) BMSC, (E) BMSC + EPO, and (F) BMSC + EPO + AMD3100 groups. Alexa Fluor 594 (Cy3) fluorescently-labeled BMSCs (red) and DAPI positive nuclei (blue) were observed at the lesion site following SCI. EPO, erythropoietin; BMSC, bone marrow-derived mesenchymal stem cell; SCI, spinal cord injury.

